# A systematic review of re-induction chemotherapy for children with relapsed high-risk neuroblastoma

**DOI:** 10.1016/j.ejca.2018.12.032

**Published:** 2019-04

**Authors:** Fiona Herd, Nermine O. Basta, Richard J.Q. McNally, Deborah A. Tweddle

**Affiliations:** a Department of Paediatric Oncology, Great North Children's Hospital, Royal Victoria Infirmary, Newcastle, NE1 4LP, UK; b Institute of Health & Society, Newcastle University, Sir James Spence Institute, Royal Victoria Infirmary, Queen Victoria Road, Newcastle upon Tyne, NE1 4LP, United Kingdom; c Wolfson Childhood Cancer Research Centre, Northern Institute for Cancer Research, Newcastle University, Level 6 Herschel Building, Brewery Lane, Newcastle upon Tyne, NE1 7RU, UK

**Keywords:** Neuroblastoma, High-risk, Relapse, Re-induction chemotherapy, Response assessment, CCLG, Children's cancer and leukaemia group, COG, Children's oncology group, CR, Complete remission, EFS, Event-free survival, HRNB, High-risk neuroblastoma, INRC, International neuroblastoma response criteria, INRG, International neuroblastoma risk group, INSS, International neuroblastoma staging system, MAT, Myeloablative therapy, MR, Mixed response, NANT, New approaches to neuroblastoma therapy, NB, Neuroblastoma, NR, Not reported, OS, Overall survival, PD, Progressive disease, PICO, Patients, intervention, comparison and outcome, PR, Partial response, RCT, Randomised controlled trial, SD, Stable disease

## Abstract

**Background:**

Despite aggressive multimodal therapy, >50% of children with high-risk neuroblastoma (HRNB) relapse. Survival after relapse is rare, and no consensus currently exists on the most effective therapy.

**Objective:**

To conduct a systematic review of the literature on effectiveness of re-induction chemotherapy in children with relapsed HRNB.

**Methods:**

Database searches were performed to identify studies looking at response to 1st line chemotherapy for children >12 months at diagnosis with first relapse of HRNB. Studies not reporting separate outcomes for HRNB patients or of refractory patients only were excluded. Two independent reviewers extracted the data and assessed study quality using a modified Newcastle–Ottawa tool.

**Results:**

Nine studies were identified fitting the inclusion criteria. All except one were single arm cohorts, and two were retrospective database reviews from single centres. One was a multicentre randomised controlled trial. All used a version of the validated International Neuroblastoma Response Criteria with 8 recording best ever response and 1 at a specified time, and 5 had central review. The proportion of relapsed patients varied from 24 to 100% with 30–93% receiving upfront myeloablative therapy. The response rate varied from 6 to 64%; however, because of heterogeneity, studies were not directly comparable, and no single treatment emerged as the most effective re-induction therapy.

**Conclusions:**

To date, there is no clear superior re-induction therapy for 1st relapse of HRNB. Randomised controlled trials with separate arms for relapsed versus refractory disease are needed to determine optimal re-induction chemotherapy to act as a backbone for testing newer targeted agents.

## Highlights

• Objective response (complete remission and partial response) is achievable in 6–64% of patients.
• Multiple drugs and regimes have been tested and show effect.
• Relapsed and refractory patients need to be studied separately.
• Currently, no one regime emerges as superior for re-induction chemotherapy at first relapse.

## 1 Background

Neuroblastoma (NB) is an embryonal tumour arising from the sympathetic nervous system originating in the adrenal gland or along the paravertebral sympathetic chain. It is a heterogeneous tumour classified into three risk groups (low, intermediate and high) depending on age, extent of disease, histology and cytogenetic abnormalities. Around 50% are high-risk neuroblastoma (HRNB) defined as unresectable or metastatic tumours with amplification of the *MYCN* oncogene in any age group or those over 18 months with metastatic disease [1].

Despite aggressive multimodal therapy, overall survival (OS) for HRNB is <50% at 5 years with most relapses (80%) occurring within 2 years of diagnosis [2]. Historically, survival after relapse was very rare. A review of relapsed stage 4 patients in the International Neuroblastoma Risk Group (INRG) database from 1990 to 2002 revealed 5-year OS of 8% and 4% for *MYCN* amplified disease [3]. An Italian retrospective review (1979–2004) found a 10-year OS for relapsed stage 4 patients of 2% [4]. UK data from a pilot epidemiological study found 3% OS at 10 years for relapsed HRNB [2]. A recent Children's oncology group meta-analysis showed a 4-year progression free survival of 6% and OS of 15% for high-risk patients enrolled on early phase trials for relapsed/refractory disease [5]. *MYCN* status, time to relapse and age have all been shown to affect length of survival after relapse with *MYCN* amplified disease progressing more rapidly, later relapse having a longer survival, and older children having a more chronic, smouldering disease [2], [3], [4], [5], [6], [7].

There is no clear consensus on optimal therapy for relapse and a lack of randomised clinical trials. A recent review on relapse therapy for HRNB summarises the rationale and data for various chemotherapeutic approaches and suggests future therapies [8] However, this is an expert review, and there is no comment on study quality or comparison of efficacy. Guidelines exist on the Children's Cancer and Leukaemia Group website, [9] suggesting a number of different chemotherapeutic regimes; they are not a systematic comparison nor do they give a preference. To avoid unnecessary toxic treatment and to optimise cure, it is essential to identify the most effective treatment in relapsed HRNB, which will also provide a backbone for testing newer targeted agents. The aim of this study was to undertake a systematic review of work, published or available in abstract form, examining effectiveness of re-induction chemotherapy in children with newly relapsed HRNB.

## 2 Methods

### 2.1 Literature search

The systematic review followed guidelines contained in the NHS Centre for Reviews and Dissemination [10]. MEDLINE, EMBASE, Cochrane CENTRAL and SCOPUS bibliographic databases from inception to December 2017 were searched using NEUROBLASTOMA and a combination of terms and their alternatives: (i) RELAPS*, (ii) HIGH RISK/STAGE 4 and (iii) TREATMENT/THERAPY/CHEMOTHERAPY/RE-INDUCTION. The reference list of a previous review paper was cross-checked [8]. Websites including clinicaltrials.gov, American Society of Clinical Oncology and Advances in Neuroblastoma Research and Solving Kids’ Cancer were also reviewed for details of any relevant studies.

### 2.2 Study selection

Randomised controlled trials (RCTs), single arm observational studies and retrospective analyses where the population studied was children with relapsed HRNB (treated on a national high-risk protocol) were included. Studies combining relapsed and refractory patients were also included. The intervention assessed was first-line chemotherapy for relapsed disease and excluded patients with >2 lines of previous therapy. The outcome measure was response rate defined by a validated tool such as the International Neuroblastoma Response Criteria (INRC) [11], [12]. An objective response was defined as complete remission (CR) or partial remission (PR). Studies were excluded if they included patients at 2nd or subsequent relapse, studied refractory disease only, were not published in English, were studies of infant patients only or were phase I studies. Relapse was defined as recurrence or progression (any new lesion, soft tissue or bone) following an initial response (including partial) to any NB therapy [11].

Three authors of studies published in abstract format were contacted via email and asked to provide full data. All declined apart from one who had a follow-up paper accepted for publication [13]. However, this article did not meet the eligibility criteria because of inclusion of heavily pre-treated patients with a median number of prior relapses of two.

One reviewer (F.H.) assessed the papers for inclusion using PICO criteria (patients, intervention, comparison and outcome) from the record title and abstract. Full papers were assessed in detail for eligibility, and any controversies were reviewed by another independent adviser (D.A.T.).

### 2.3 Data extraction

Study characteristics and results were extracted by two independent reviewers (F.H. and N.O.B.) using a specially designed proforma (Supplementary Table 1). Trial methodology/quality was assessed subjectively and using a modified version of the Newcastle Ottawa Tool [14] for cohort studies after review of options [15]. A third independent adviser (D.A.T.) reviewed any discrepancies between the two reviewers. A cut-off of 60% was chosen for the proportion of relapsed patients and proportion of patients having initial high-dose myeloablative therapy (MAT) and autologous stem cell rescue for the study to be deemed a representative sample, since this research was focussed on first relapse of patients treated on a previous high-risk protocol.

## 3 Results

**Fig. 1:**
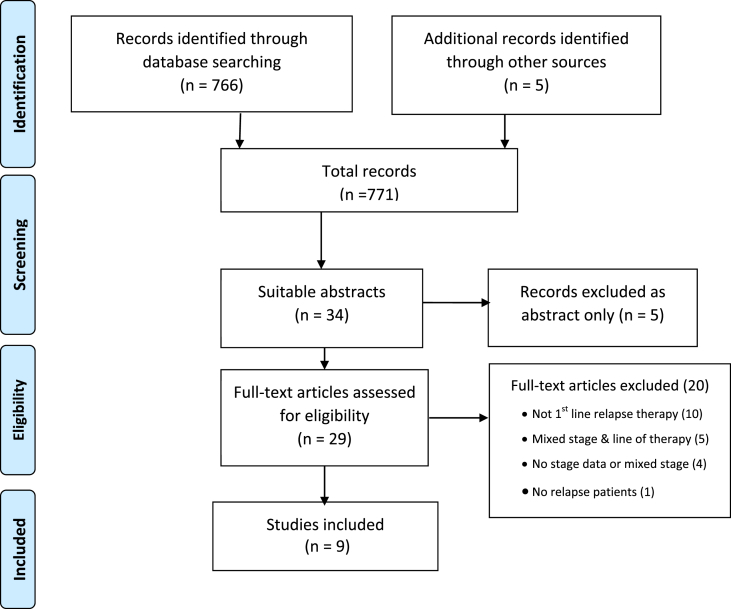
Flow diagram of included studies.

Electronic searching yielded 766 records, and an additional five other records were identified making a total of 771 records. Thirty-four full-text articles were assessed for study eligibility, and nine studies met the inclusion criteria. Most exclusions were because of all stages of disease being included without subgroup analysis or patients receiving more than two previous lines of chemotherapy (Fig. 1).

The characteristics of the nine included studies [16], [17], [18], [19], [20], [21], [22], [23], [24] are detailed in Table 1. The studies were undertaken between 1999 and 2015 and published between 2003 and 2017. Six were single arm, prospective studies with small cohorts of relapsed and refractory patients (25–40 patients) [17], [18], [19], [21], [22], [23]. One study had three different treatment arms depending on whether the patient had a central line *in situ,* and then a dose escalation was performed after the toxicity was deemed acceptable [22]. Two studies were single centre retrospective database reviews [16], [20]. Of the other studies, four were single arm, prospective multicentre studies within one country [17], [19], [22], [23], and two were European multicentre studies [18], [21]. Only one study [24] was a randomised controlled study.

**Table 1:** Summary of included studies and their characteristics.

Study	Type	Patient no.	Aim/intervention	% Relapsed	% MAT
Ashraf 2013 [16]	Retrospective database review in single centre	27	Describe response, survival and toxicity of cyclophosphamide and topotecan in children with 1st relapse of NB	96	93
Bagatell 2011 [17]	Prospective single arm cohort in COG centres	27^a^	Determine response rate of irinotecan and temozolomide in relapsed/refractory NB	77	NR
Di Giannatale 2014 [18]	Prospective single arm cohort in Europe	38	Assess objective response rate of 2 cycles of topotecan & temozolomide chemo	66	61
Garaventa 2003 [19]	Prospective single arm cohort in Italy	25	Evaluate anti-tumour activity and tolerability of topotecan/vincristine/doxorubicin) in children with advanced NB	24	52
Kushner 2010 [20]	Retrospective database review in single centre	30^a^	Assess likelihood of response to high dose cyclophosphamide/topotecan/vincristine	100	70
Rubie 2006 [21]	Prospective single arm cohort in Europe	25	Determine response rate of NB to temozolomide	60	64
Simon 2007 [22]	Prospective single arm cohort in Germany	40	Trial of topotecan & etoposide in the treatment of patients with relapsed HRNB	100	30
Simon 2007 [23]	Prospective single arm cohort in Germany	33^a^	Trial of topotecan/cyclophosphamide/etoposide in the treatment of patients with HRNB	100	52
Mody 2017 [24]	Randomised Control Trial in COG centres	35	Comparison of temozolomide & irinotecan chemotherapy with additional temsirolimus or dinutuximab in 1st relapse of HRNB	56 & 53	50 & 59

MAT, myeloablative therapy with autologous stem cell rescue; NR, not reported; NB, neuroblastoma; HRNB; high-risk neuroblastoma; COG, Children's oncology group.

a n = number of participants from the entire cohort in eligible sub group(s).

### 3.1 Evaluation of studies meeting inclusion criteria

All studies included patients with HRNB ranging from three studies [20], [22], [23] comprising all relapsed patients to just 24% in one study [19]. The percentage of patients who had received MAT with stem cell rescue as prior treatment varied from 30 to 93% but was not reported in one study [17]. Only one study documented prior use of immunotherapy [24]. In some studies, only certain subgroups of the total study cohort were suitable for inclusion: one study [17] split their cohort into two strata with 28 patients in stratum 1 who had measurable disease, but only 50% of these were stage 4 at diagnosis and others stage 1–3. Not all of these non-stage 4 patients had *MYCN* amplification, and therefore not all were defined as high-risk patients. Stratum 2 had 27 patients with disease evaluable by bone marrow or meta-iodo benzyl guanidine (mIBG) only, and all were high-risk at diagnosis so only this arm was included. Another [20] reported a total of 126 patients split into four groups—new recurrence, primary and secondary refractory and progressive disease. Only the subgroup of new recurrence (30 patients) was included. A further study [23] included a total of 44 patients split into two cohorts: 33 had new recurrences and were included, and 11 were newly diagnosed patients, so were excluded.

### 3.2 Response assessment

**Fig. 2:**
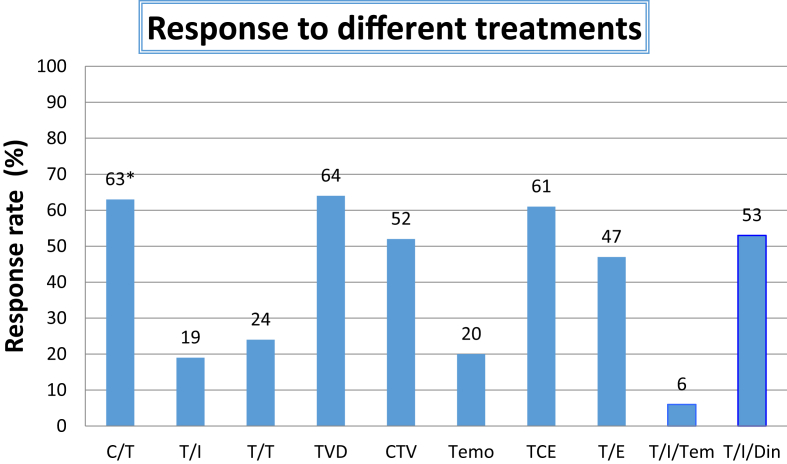
Bar chart displaying the objective response rate (complete remission & partial remission) to different chemotherapeutic strategies detailed in Table 1. C/T, Cyclophosphamide/Topotecan; T/I, Temozolomide/Irinotecan; T/T, Topotecan/Temozolomide; TVD, Topotecan/Vincristine/Doxorubicin; CTV, Cyclophosphamide/Topotecan/Vincristine; Temo, temozolomide TCE, Topotecan/Cyclophosphamide/Etoposide; T/E, Topotecan/Etoposide; T/I/T, temozolomide/Irinotecan/Temsirolimus; T/I/D, temozolomide/Irinotecan/Dinutuximab. *Mixed response is included in response

Table 2 provides a description of response assessments performed in each study and outcome. All studies described an objective response rate to treatment using validated criteria, although for two, this was a secondary outcome [22], [23]. All studies used the INRC [11], although one study [16] defined it as the New Approaches to Neuroblastoma Therapy criteria, which is a modified version of INRC. Most described best ever response, but one used response at a pre-defined time point [17]. Eight of the studies defined response as CR and PR, but one study included mixed response (MR) [16]. Response varied from 6 to 64% (Fig. 2).

**Table 2:** Summary of response assessment in each study.

Study	Drug	Timing of response assessment	Response rate (%)
Ashraf 2013 [16]	Topotecan/cyclophosphamide	Best	63^a^
Bagatell 2011 [17]	Temozolomide/irinotecan	After 3 cycles	19
Di Giannatale 2014 [18]	Temozolomide/topotecan	Best	24
Garaventa 2003 [19]	Topotecan/vincristine/doxorubicin	Best	64
Kushner 2010 [20]	Cyclophosphamide/topotecan/vincristine	Best	52
Rubie 2006 [21]	Temozolomide	Best	20
Simon 2007 [22]	Topotecan/etoposide	Best	47
Simon 2007 [23]	Topotecan/cyclophosphamide/etoposide	Best	61
Mody 2017 [24]	Temozolomide/irinotecan + temsirolimus + dinutuximab	Best	6 53

a Denotes that the response includes mixed response.

### 3.3 Quality assessment

Study quality is shown in Table 3. Two studies were not as representative of the desired patient group because of a low percentage of relapsed patients in the cohort and the low number who had received previous high-dose chemotherapy treatment, respectively [19], [23]. All studies described treatment exposure and adherence adequately. All studies used a validated tool for response assessment with five studies having central review of response, but little information was provided about blinding of reviewers [17], [18], [19], [21], [24]. Since the primary outcome was response, there was no requirement for long follow-up, and all patients were available for assessment. Two studies were retrospective single-centre studies, so were not representative of a wide cohort. Because only one study [24] was a randomised controlled study, a formal tool for quality assessment was not used, but appropriate methods of randomisation were used, and the two arms were relatively similar for important prognostic characteristics including *MYCN* status. The only difference was the percentage with bone marrow disease, which was 33% in the temsirolimus arm and 76% in the dinutuximab arm.

**Table 3:** Quality assessment^a^ of studies.

Study	Selection				Outcome			Other	Composite score
	Representative		Ascertainment of exposure	Outcome not present at start	Assessment of outcome		Follow-up		
	>60% relapse	>60% MAT			Validated tool	Central review			
Ashraf 2013 [16]	✓	✓	✓	✓	✓		✓	Retrospective single centre	6
Bagatell 2011 [17]	✓	NR	✓	✓	✓	✓	✓	Alternative definition of high risk	6
Di Giannatale 2014 [18]	✓	✓	✓	✓	✓	✓	✓		7
Garaventa 2003 [19]			✓	✓	✓	✓	✓		5
Kushner 2010 [20]	✓	✓	✓	✓	✓		✓	Retrospective single centre	6
Rubie 2006 [21]	✓	✓	✓	✓	✓	✓	✓		7
Simon 2007 [22]	✓		✓	✓	✓		✓	Alternative definition of high risk; Variation in treatment delivered	5
Simon 2007 [23]			✓	✓	✓		✓	Alternative definition of high risk	4
Mody 2017 [24]			✓	✓	✓	✓	✓	Randomised trial	5+

MAT, myeloablative therapy with autologous stem cell rescue.

✓ denotes study meets the criteria, NR, not reported.

Alternative definition of high risk refers to [1]: The US groups including children over 18 months with localised unresectable tumours & unfavourable histology group as high risk, whereas in the UK/rest of Europe, these groups are classed as intermediate risk [2]; The German studies include localised resectable tumours with *MYCN* amplification as high risk when others do not and exclude the 12–18 months with metastatic disease but no *MYCN* amplification from their high-risk group.

a Quality assessed using modified Newcastle Ottawa Scale [13].

## 4 Discussion

Historically, trials undertaken in relapsed neuroblastoma describe a very heterogeneous patient group. Often, relapses of all stages of disease are included as well as inclusion of a combination of refractory and relapsed patients. This review focussed on relapsed HRNB. Relapsed patients respond differently compared with refractory patients [19], [20], with the latter less likely to show an objective response to chemotherapy but with a longer time to progression and better OS [25]. One study had just 24% relapsed patients (6 patients) [19]. However, the remaining studies comprised predominantly relapsed patients with three having an entirely relapsed cohort. *MYCN* amplification is associated with a poorer OS and a shorter survival time post relapse [2], [3], [26], [27]. Older patients without *MYCN* amplification often have a more chronic, smouldering disease with longer survival [6]. Therefore, age distribution and presence of *MYCN* amplification is important in interpretation of results. Seven studies reported the proportion of patients with *MYCN* amplification, and this ranged from 10 to 38%. The percentage of stage 4 patients with *MYCN* amplification is around 30% [28], and in high-risk disease, this is slightly higher because of localised *MYCN* amplified tumours being included. A pilot study of relapsed patients found the rate of *MYCN* amplification to be 42%, [2] so the proportions reported in these studies are slightly lower than expected. Relapses occurring earlier after diagnosis are associated with a shorter length of survival [3], [27], but the effect of other prognostic factors (age, *MYCN* amplification and time to relapse) were not reported in studies included in this review.

Neuroblastoma staging is standardised worldwide using the International neuroblastoma staging system and INRG criteria [1], [11]. However, the decision to treat patients on national high-risk protocols varies, e.g. in most of Europe, HRNB is defined as patients over 12 months with metastatic disease, *MYCN* amplified localised unresectable disease and infants with *MYCN* amplified metastatic disease [1]. However, German protocols include *MYCN* amplified resectable tumours on their high-risk protocols [29], [30] but exclude metastatic disease without *MYCN* amplification diagnosed between 12 and 18 months. Both German and North American protocols include children over 18 months with unresectable localised tumours showing International Neuroblastoma Pathology Classification unfavourable histology with or without unfavourable genetics. Thus, patient groups may be slightly different, potentially affecting outcome and response.

Not all centres use MAT and stem cell rescue in upfront treatment of HRNB. Refractory patients are less likely to have had previous MAT or immunotherapy. Response to relapse therapy may be different in those who have had prior MAT or immunotherapy. The included studies varied with respect to the proportion who had received prior MAT with four studies not having the desired 60% of patients having this treatment [19], [22], [23], [24]. Studies also lacked description of on-going therapy after the regimen reported in the papers. Some studies continued until disease progression, and others were for a prescribed number of cycles, with the aim of obtaining a response and then continuing to consolidation therapy (although this was not described). In most therapeutic phase III trials, OS and event-free survival (EFS) are the primary outcome measures and with the aim of ultimately improving OS of relapsed HRNB. However, because of the low survival and lack of standardised therapy post re-induction therapy and that many of the included trials did not report EFS or OS, comparison was impossible. Time to progression could not be used because of the varying treatment strategies given after the investigative treatment; therefore, response rate was the only suitable outcome measure but may not equate to survival.

The widespread use of standard definitions of response allows confidence that studies are comparing similar outcomes although one study included MR [16]. In 2012, revisions were made to the INRC to use Response Evaluation Criteria in Solid Tumours for response assessment of primary and metastatic soft tissues sites and to classify bone marrow involvement of ≤5% as minimal disease allowing these patients to be eligible for a PR depending on other site responses. Urinary catecholamine levels were removed from response assessment [12]. These changes arose with the advent of more modern imaging technology and a lack of sensitivity and specificity from the older techniques. Therefore, we recognise that even though validated INRC was used throughout the included studies, they have limitations and are not now the gold standard reassessment tool. The use of central review in five of the studies helped to reduce bias in response categorisation.

Whilst direct comparisons are limited, it was interesting that one study [21] reported a response rate (RR) of 20% to temozolomide alone, and two other studies [17], [18] assessing temozolomide with the addition of a second agent such as irinotecan or topotecan were very similar (19 & 24%), yet the RCT study [24] showed that the temozolomide/irinotecan and temsirolimus arm had a disappointing response rate of just 6%. It is not clear why it was so much lower in this group. There were no major differences in the patients included in these studies with a mix of relapsed and refractory patients mostly with metastatic disease. It may be worth noting that the three regimes with a >60% response rate contained topotecan [16], [19], [23]. In the United States, topotecan has been moved into frontline treatment because of its efficacy at relapse in the hope that it could reduce relapse rate [31].

A double-blind RCT is the gold standard method for comparison of therapeutic efficacy. A large phase II RCT, comparing cyclophosphamide and topotecan with topotecan alone demonstrated a slight, but non-significant improvement in response in the combination arm, which did not translate into improvement in survival [26]. This study was excluded because of inclusion of non–high-risk patients, without separate high-risk analysis [30]. A pilot study of temozolamide, irinotecan, rapamycin and dasatanib (RIST) in relapsed and refractory neuroblastoma patients published in abstract form showed an objective major response rate (CR and PR) of 71%, and a larger trial is now underway [32]. The on-going European BEACON trial will provide additional information on potentially suitable backbone chemotherapy [33]. A non-randomised cohort study found no benefit of adding bevacizumab to temozolomide and irinotecan in relapsed and refractory patients, and it will be important to see if this is confirmed by the BEACON study too [13]. This review is limited by the strict inclusion criteria, which were chosen at the outset in order not to replicate previous work and with the aim to identify the best treatment for patients at first relapse with the intention of cure rather than palliation. However, the authors recognise that this has led to exclusion of several other regimes [26], [34], [35].

The current review is subject to publication bias since all included studies were published in peer-reviewed journals despite searching for unpublished literature. Study heterogeneity with regard to risk group and previous treatments made formal quality assessment/scoring difficult and direct comparison of results and meta-analysis impossible.

## 5 Conclusion

Children with relapsed high-risk neuroblastoma show response to a variety of chemotherapy agents. However, patient and prior treatment heterogeneity in published studies precludes determination of the most effective re-induction strategy for children with relapsed high-risk neuroblastoma. International and perhaps worldwide RCTs in patients having similar upfront treatments powered to look at individual subgroups (relapsed versus refractory, *MYCN* amplification status) are required to determine the ideal backbone upon which to test novel targeted agents to try and cure more children with relapsed high-risk neuroblastoma.

## Conflict of interest statement

None declared.
