# Supplementary material for: A systematic review of re-induction chemotherapy for children with relapsed high-risk neuroblastoma
Source: Eur J Cancer. 2019 Apr;111:50–8. doi: 10.1016/j.ejca.2018.12.032 (PMC6458963; doi:10.1016/j.ejca.2018.12.032)
Supplement: Multimedia component 1 [file mmc1.docx]

| **Supplementary Table 1 - DATA EXTRACTION FORM** | | | | |
| --- | --- | --- | --- | --- |
| First Author (year of publication) | |  | | |
| Journal name | |  | | |
| Type of study | retro / prospective |  | | |
|  | cohort / RCT |  | | |
| Phase | |  | | |
| Sponsor | |  | | |
| Aim | |  | | |
| Location | |  | Single Centre Y / N | |
| Date of study | |  | | |
| Duration (months) | |  | | |
| Population | inc criteria | histologically confirmed Bx |  | |
|  |  | BM positive |  | |
|  |  | raised urine cats |  | |
|  |  | other |  | |
|  | exc criteria |  | | |
|  | stage |  | | |
|  | rel/ref |  | | |
|  | upfront therapy |  | high dose Y / N | |
|  | age (years) |  | | |
|  | timing of relapse |  | | |
| Intervention | drug |  | | |
|  | dose |  | | |
|  | route |  | | |
|  | timing |  | | |
|  | No of cycles |  | | |
|  | additional therapy |  | | |
| Comparison | drug |  | | |
|  | dose |  | | |
|  | route |  | | |
|  | timing |  | |  |
|  | no. of cycles |  | |  |
| Other treatment (consolidation) | |  | | |
| Follow up (months) | |  | | |
| Outcomes measured | response rate (which criteria)  central review (Y/N) | INRC | |  |
|  |  | NANT | |  |
|  |  | RECIST | |  |
|  | PFS time (months) |  | | |
|  | OS/EFS (months) |  | | |
|  | tolerability / AE |  | | |
| No of patients | planned |  | | |
|  | enrolled |  | | |
|  | completed |  | | |
|  | follow up |  | | |
| Results | |  | | |
| Quality – using modified Newcastle Ottawa Scale | |  | | |
| Conclusions | |  | | |
